# Supplementary figures and images for: Parvovirus nonstructural protein 2 interacts with chromatin-regulating cellular proteins
Source: PLoS Pathog. 2022 Apr 8;18(4):e1010353. doi: 10.1371/journal.ppat.1010353 (PMC9020740; doi:10.1371/journal.ppat.1010353)

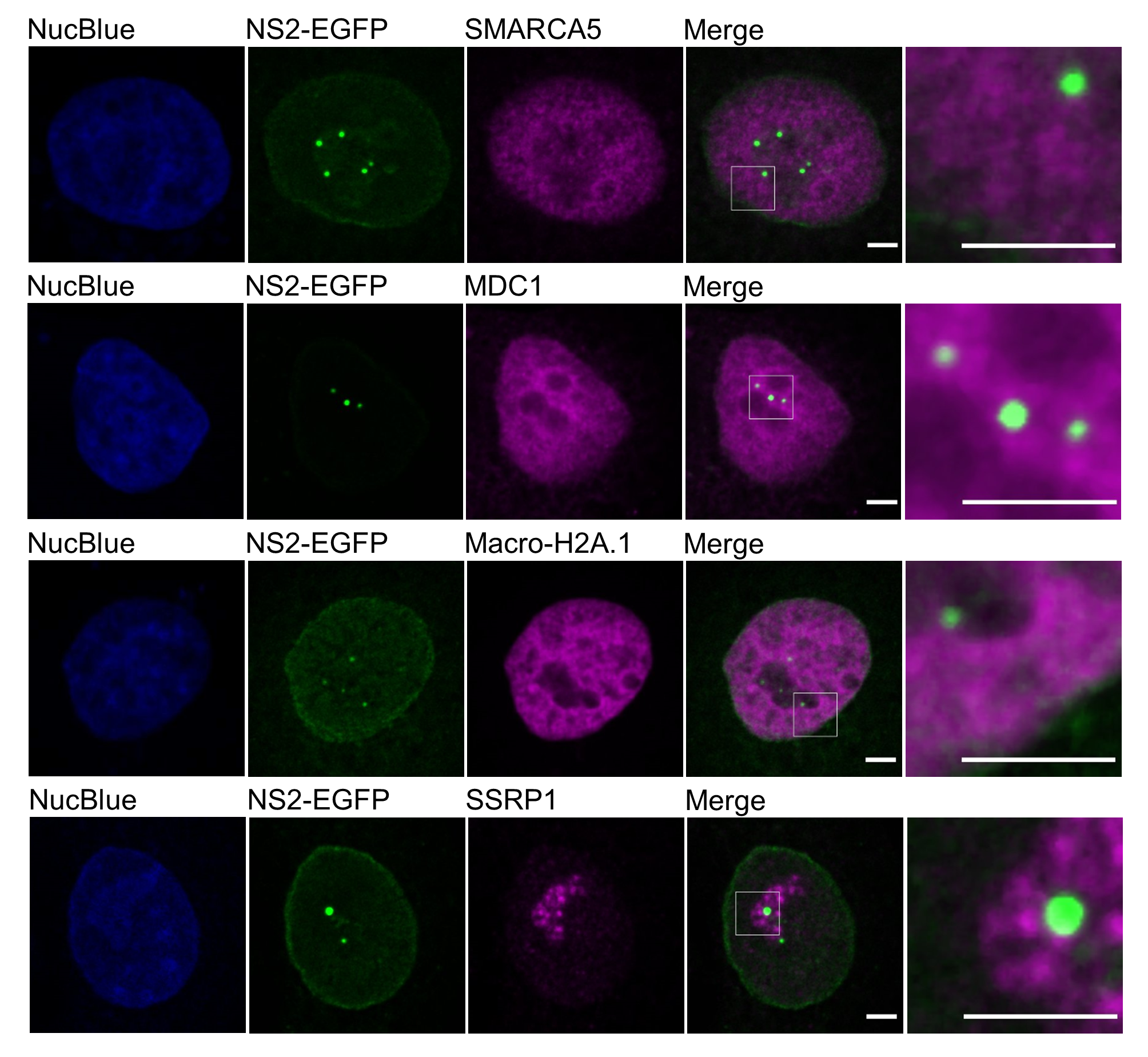

Supplement: S1 Fig — Representative confocal microscopy images showing the localization of NS2-EGFP (green) together with selected BioID hits SMARCA5, MDC1, macro-H2A.1, and SSRP1 (magenta) in HeLa cells at 24 hpt with NucBlue stained nuclei (blue). Merged images show the localization of NS2-EGFP with the BioID-identified interactors. Close-up images of selected regions are shown (right). Scale bars, 3 μm. (TIF) [file ppat.1010353.s001.tif]

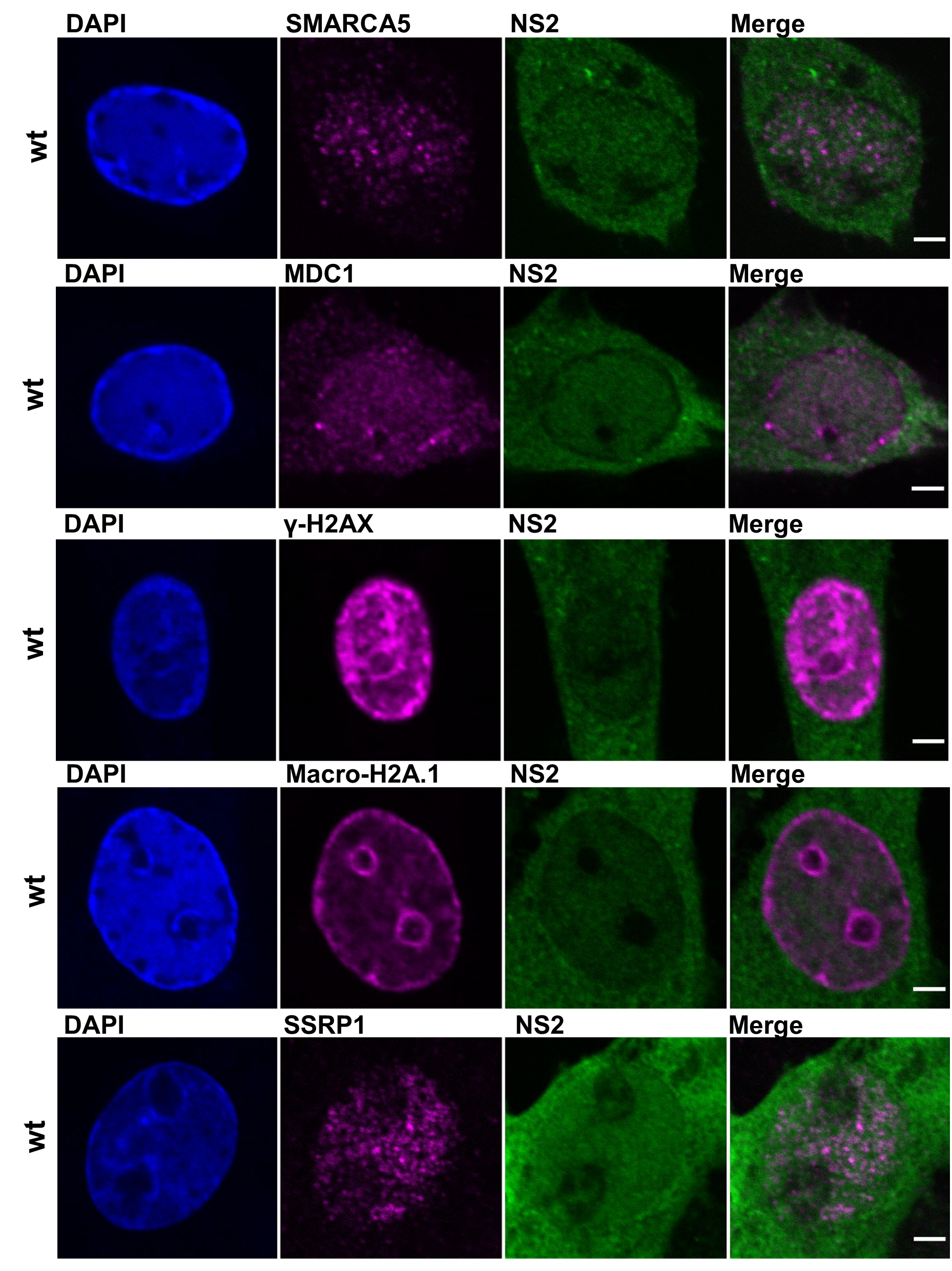

Supplement: S2 Fig — Representative confocal microscopy images showing the localization of NS2 (green) identified by NS2 antibody together with selected BioID hits including SMARCA5, MDC1, γ-H2AX, macro-H2A.1, and SSRP1 (magenta) in NLFK cells at 24 hpi with DAPI-stained nuclei (blue). Merged images show the localization of NS2 with the NS2 interactors. Scale bars, 3 μm. (TIF) [file ppat.1010353.s002.tif]

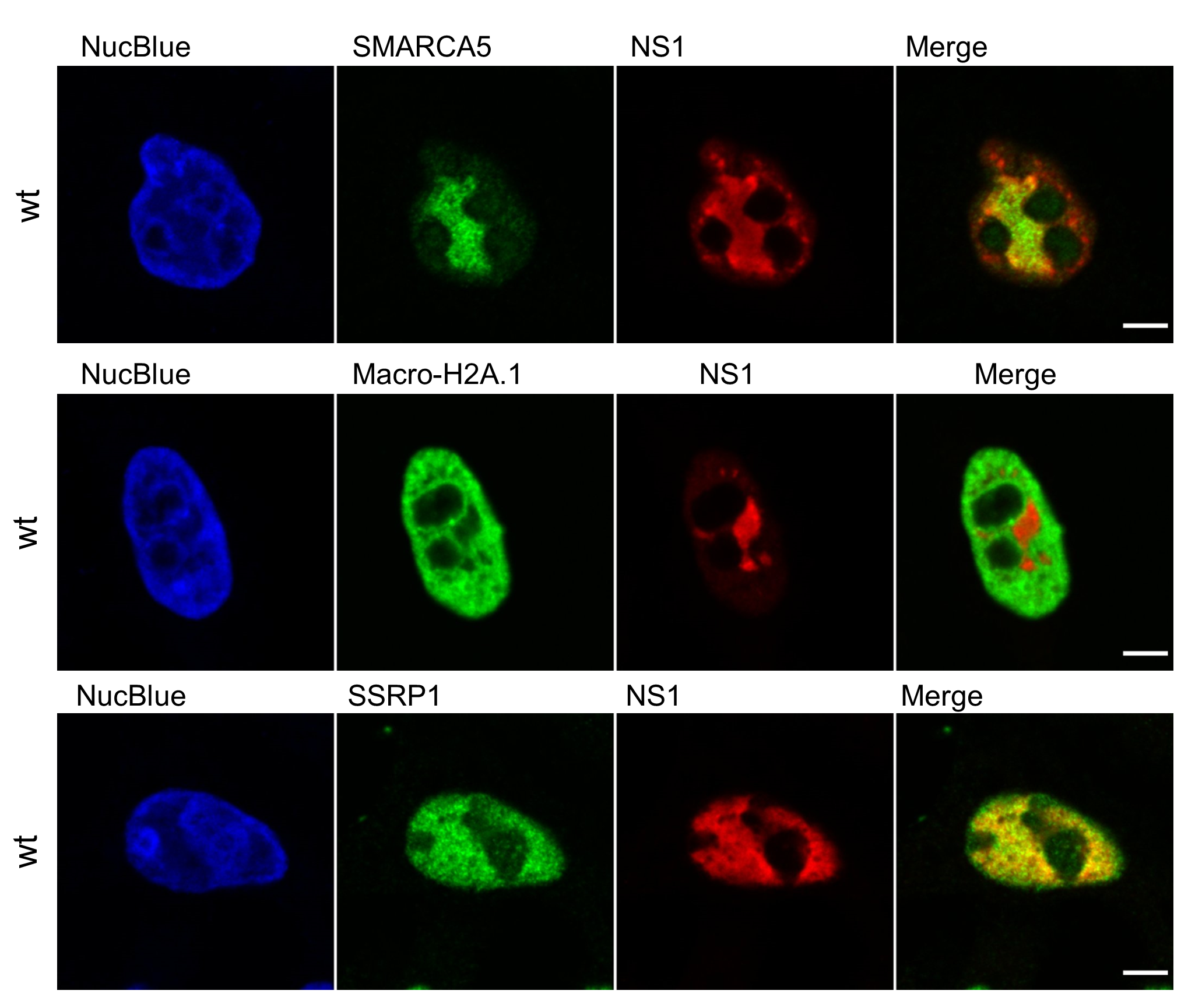

Supplement: S3 Fig — Representative confocal microscopy images showing the localization of NS1 (red) together with selected NS2 BioID hits including SMARCA5, Macro-H2A.1 and SSRP1 (green) in NucBlue-labeled nucleus (blue) of wt CPV-infected in HeLa cells at 24 hpi. Merged images show the localization of replication protein NS1 with the BioID-identified interactors of NS2. Scale bars, 5 μm. (TIF) [file ppat.1010353.s003.tif]

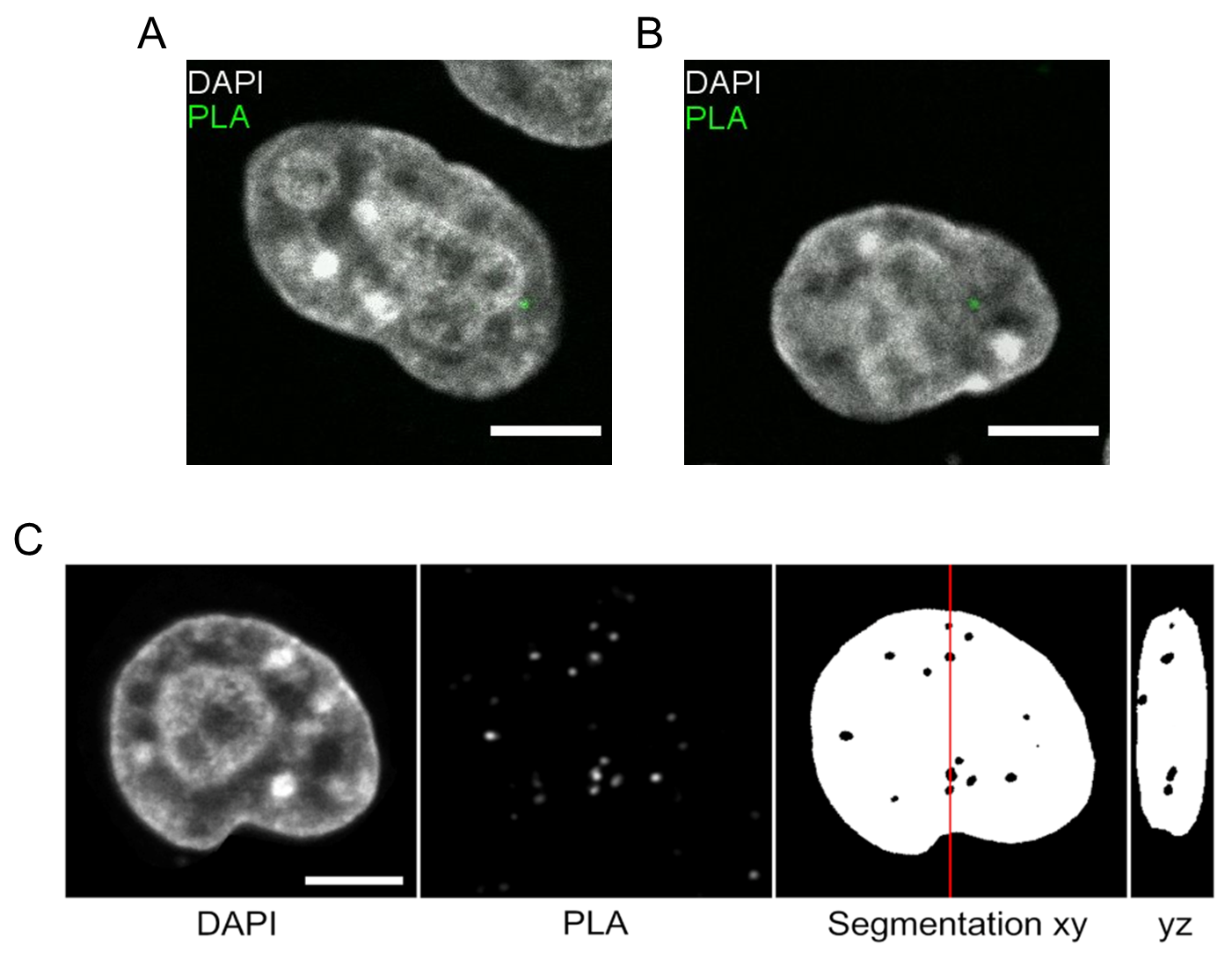

Supplement: S4 Fig — Images of technical controls with either (A) both PLA probes without antibodies or (B) both PLA probes with only GFP antibody. Representable confocal microscopy sections show PLA signals (green) and the nuclei of HeLa cells stained with DAPI (gray). (C) The image shows confocal sections of nuclear DNA stained by DAPI (gray, left) and PLA signals (white, middle). The segmented nucleus is shown by xy- and yz-slices (white, right) in addition to PLA signals (black). The yz-slice is taken along the line shown in red color. Scale bars, 5 μm. (TIF) [file ppat.1010353.s004.tif]

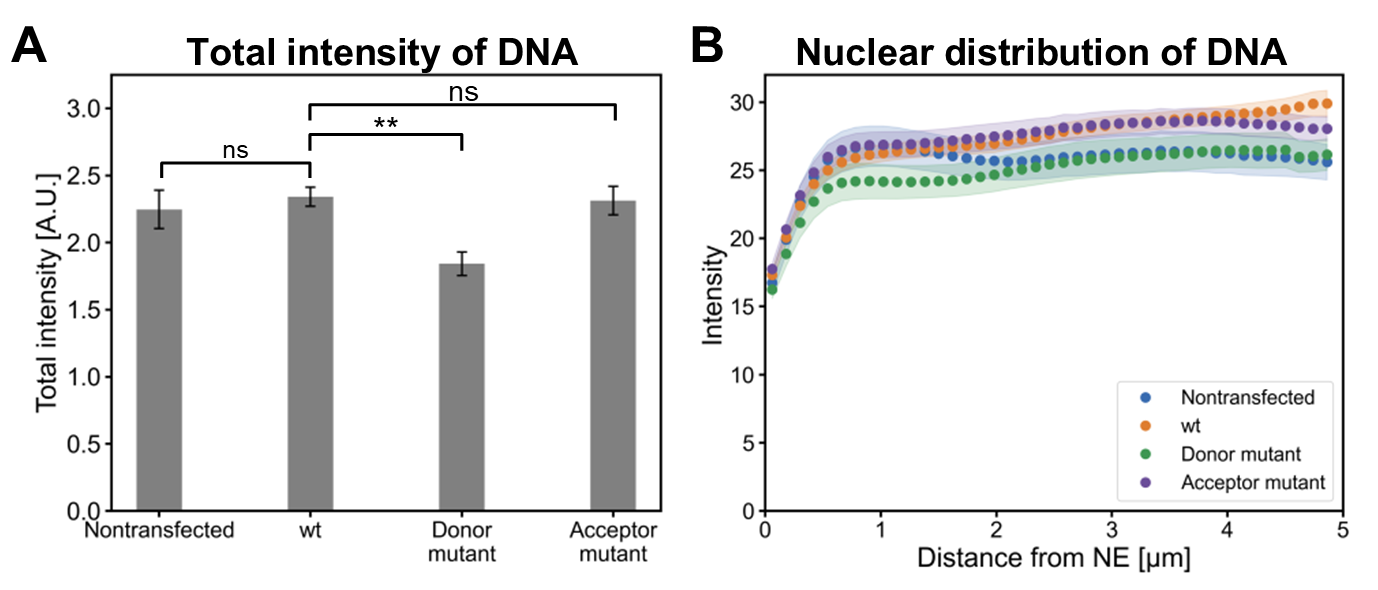

Supplement: S5 Fig — (A) Total fluorescence intensities of DAPI-stained DNA in nontransfected, wt and NS2 mutants transfected HeLa cells (24 hpt) (n = 27). (B) Localization of DNA as a function of increasing distance from the NE in transfected cells (n = 29). The error bars show the standard error of the mean. Statistical significances were determined using Dunnett’s multiple comparison test. The significance values shown are denoted as ** (p<0.01), * (p<0.05) or ns (not significant). (TIF) [file ppat.1010353.s005.tif]

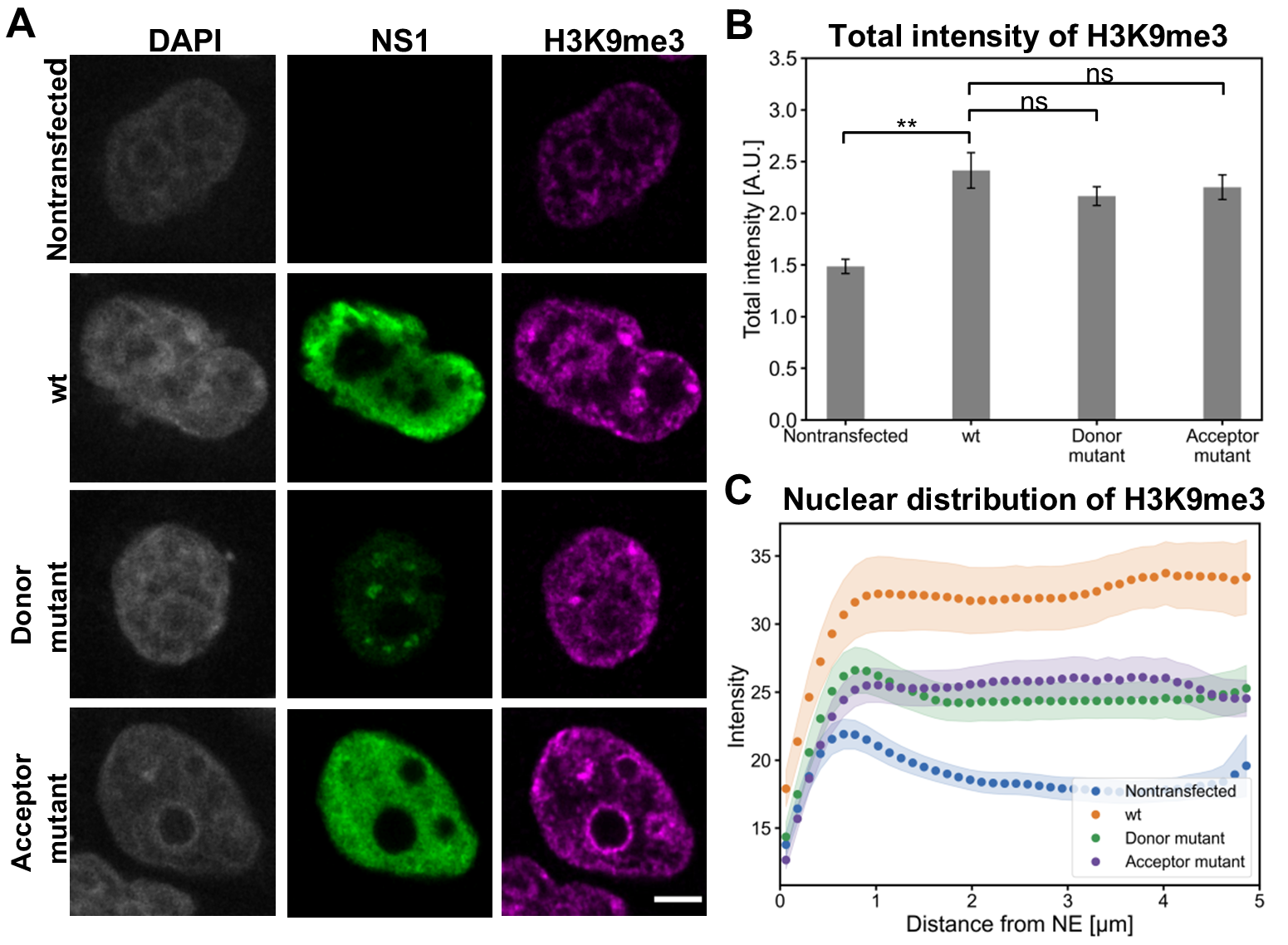

Supplement: S6 Fig — (A) Representative confocal images of heterochromatin marker H3K9me3 (magenta), NS1 (green), and DAPI (gray) distribution in nontransfected HeLa cells, and cells transfected with wt, NS2 donor and acceptor mutants at 24 hpt. (n = 25). (B) Fluorescent intensities and (C) nuclear localization of H3K9me3 in nontransfected and transfected cells. The error bars show the standard error of the mean. Statistical significances were determined using Dunnett’s multiple comparison test. The significance values shown are denoted as ** (p<0.01), or ns (not significant). Scale bars, 5 μm. (TIF) [file ppat.1010353.s006.tif]
