# Supplementary material for: Parvovirus nonstructural protein 2 interacts with chromatin-regulating cellular proteins
Source: PLoS Pathog. 2022 Apr 8;18(4):e1010353. doi: 10.1371/journal.ppat.1010353 (PMC9020740; doi:10.1371/journal.ppat.1010353)
Supplement: Attachment — Submitted filename: Revision letter final.pdf [file ppat.1010353.s011.pdf]

PLOS Pathogens
Editors
Matthew D Weitzman
Karl Münger
Kasturi Haldar
Michael Malim

December 17th, 2021

Dear Editors,

Please find enclosed the revised manuscript "*Parvovirus nonstructural protein 2 interacts with chromatin-regulating cellular proteins*" by Salla Mattola et al.

We greatly appreciate the opportunity given to revise our manuscript. We have now addressed all the points raised by the reviewers as outlined in our response letter. Based on their suggestions, appropriate changes have been introduced to the manuscript. We trust we have been able to address their concerns and hence to improve our work.

We hope our manuscript is now suitable for publication in PLOS Pathogens.

Yours sincerely,

Maija Vihinen-Ranta et al.

Department of Biological and Environmental Science
University of Jyväskylä, Finland
Phone: +358 400 248 118
Email: maija.vihinen-ranta@jyu.fi

Response to the reviewers' comments

Part II – Major Issues: Key Experiments Required for Acceptance

Reviewer #1

BioID experiments show direct NS2 interactions with cellular proteins involved in, among other things, chromatin organization and the DNA damage response. Proximity-dependent ligation experiments verify some of the most notable interactions, and localization of expressed NS2 shifts during infection. These experiments are well done and provide clues to potential roles for NS2 during infection. However, the authors claim that "our results provide unique insights into novel roles of NS2 in controlling chromatin remodeling and DNA damage response necessary for successful viral replication." This remains unsubstantiated.

We are deeply indebted to all Reviewer #1 for his/her constructive comments and invaluable help in improving our manuscript.

Appreciating the Reviewer's suggestion, we have modified the claim in the sentence starting *"*our results provide... *"*. (Abstract, last sentence).

Major criticisms:

Most importantly, it is imperative to examine NS2 and NS1 expression in the donor and acceptor mutants being studied. Donor mutations often lead to cryptic or spurious splicing, and the products generated from the donor mutant NS region are unknown. This mutant may not generate NS2 at all, or perhaps it generates an NS2 fragment, or perhaps it splices in frame into the NS1 coding region generating a chimeric mutant protein. This knowledge is essential for further interpretation. Also, does the splice acceptor mutation also prevent wt NS2 production as might be expected? If so, and as it behaves much like wild-type, what would that say about the requirement of NS2 during infection? I suspect that performing such experiments may be hampered by the lack of available reagents; however, it remains very difficult to draw conclusions about the role of NS2 with these uncertainties.

Our response

In this study we have used NS2 splice donor and splice acceptor mutants (G533A, A2003T) that were previously described in a publication from the Parrish group (Wang et al. 1998). Those were based on mutants previously described as influencing the expression of NS2 of the MVM virus (Carter et al. 1992, Naeger et al. 1993). Although there might be differences in the sequences and perhaps in the transcription scheme of MVM and CPV, the mutations block the expression of intact CPV NS2 (Wang et al. 1998).

The original publication clearly showed that viral capsid protein (VP1/VP2), capsid and viral DNA production were all significantly decreased in NS2 splice donor mutant transfected but not in splice acceptor mutant transfected cells. The production of capsid protein in the splice donor mutant transfection was less than 1/10 of the wt and other acceptor mutants examined. At the same time, transfection with splice acceptor mutant led to production almost equal to wt CPV. This shows that some key functions essential for viral replication are most likely removed from the NS2 splice donor mutant. In the original paper from Colin Parrish`s group (Wang et al 1998) NS2 mutants were produced by introducing termination codons into the NS2 coding sequence of the infectious clone. In NS2 mutants, termination codons located to a site upstream of the common NS1/2 splice donor or downstream of the NS2-specific splice acceptor. Depending on the site of the termination codon most likely different sized NS2 proteins are produced. Although, characterization of mutants by immunoprecipitation analyses (antibodies against the NS2 C-terminus), showed that both mutants were unable to produce intact NS2 protein (Wang et al 1998), the NS2 splice donor mutation likely leads to drastically shorter protein than the splice acceptor. The original data showed that although both splice donor and acceptor mutants were not intact the deficiency of acceptor mutant did not dramatically affect its function whereas the splice donor mutation significantly changed the function of the NS2.

We therefore believe that the NS2 splice donor mutant is a very useful tool to support our studies of NS2 function. We have clarified our statement of the impact of NS2 mutation on the progression of infection and renewed all Figures of NS2 mutants (Figures 7, 8, 9, Results, pages 14-18, lines 360-376).

Refs
Wang et al. 1998, https://pubmed.ncbi.nlm.nih.gov/9454701/
Cater et al. 1992, https://pubmed.ncbi.nlm.nih.gov/1385828/
Naeger et al. 1993, https://pubmed.ncbi.nlm.nih.gov/8419637/

The experiments looking at NS2 effects related to DDR proteins is confounded by the uncertainty of the mutants used, but in addition, as the authors point out (line 395), by potential differences in mutant viral replication. If this is the case, any effects attributable to NS2 that can be made are at best secondary in nature. Viral replication of parvoviruses induces a DDR and the authors demonstrate that; however, the authors are trying to make the case that NS2's role directly affecting the DDR plays a gateway role facilitating infection. This is an attractive possibility; however, notwithstanding NS2's interaction with DDR proteins here identified, these experiments do not illuminate roles in this process beyond NS2's anticipated effect on replication – and even this is muddied by lack of characterization of the mutants. Determining the levels of replication of the mutants is an important aspect that may help to begin to resolve this issue.

The studies of David Pintel´s group demonstrated that the formation of viral replication centers is adjacent to sites of DDR (Majumder et al. 2018). Here, we show that NS2 is associated with DDR proteins.

As discussed above, the inefficient replication of the NS2 splice donor mutant was demonstrated in the studies of Wang et al. (1998). They demonstrated a significant decrease in capsid protein (VP1/VP2), capsid and viral DNA production in NS2 splice donor mutant transfected cells but not in the splice acceptor transfected cells.

However, as the reviewer said, the exact mechanism of potential NS2 interaction with DDR proteins, its role as direct or indirect mediator between virus and DDR machinery, and the importance of this role for progression of infection remains to be studied and described. The manuscript has been modified accordingly.

Refs.
Majumder et al. 2018, https://pubmed.ncbi.nlm.nih.gov/30028293/
Wang et al. 1998, https://pubmed.ncbi.nlm.nih.gov/9454701/

The experiments describing the correlation of NS1 levels with the DDR protein levels is very confusing. Is NS1 being used as a proxy for replication? The argument that differences in correlation show the requirement of NS2 for progression of the DDR is not convincing, especially in the absence of replication data. Also, discordance of the correlation for the splice acceptor mutant seems inconsistent with its ability to replicate well. These results need additional explanation.

We truly appreciate these truthful comments and they helped us to clarify the experiment and data analysis.

The NS1 labeling has been used as a marker of viral replication centers to verify the infection (a sentence added to results page 8, line 94). NS1 staining allows verification of the splice donor mutant-induced decrease in the size of viral replication centers (see new Figure 9). Previous MVM studies have also shown that NS1 is localized adjacent to DNA damage sites and has a role during the localization of the viral genome to these sites (Majumder et al. 2018).

To analyze further the role of NS2 on nuclear distribution of DNA damage proteins we monitored the localization of γ-H2AX and MDC1 in the periphery of replication centers identified by NS1 labeling. We focused on the periphery of replication centers that are typically located close to the NE and around the nucleoli at late stages of infection. In here we have replaced the correlation analysis with the more suitable fluorescent intensity line profile analysis. This analysis demonstrated that γ-H2AX and MDC1 were both accumulated close to the edges of the viral replication centers in wt and splice acceptor mutant transfected cells clearly less in the splice donor mutant transfected cells.

The whole chapter (page 15-16, lines 400-437), figure 8 and figure legend have been renewed.

Refs.
Majumder et al. 2018, https://pubmed.ncbi.nlm.nih.gov/30028293/

Major Issues, Reviewer #2:

We are deeply indebted to the Reviewer 2 for her/his competent comments.

1. Fig. 1: Some confocal images are of low quality; for example, the colocalization of NS2 and nucleolin in Fig. 1C is not very convincing. Also, what statistical analysis was used in Fig. 1A?

Figure IC has been replaced by better representative figure. It is named 1D in the final figure.

In the Fig. 1A, the student's t-test was used to analyze the values at each time points to see if their difference was statistically significant. There was no statistically significant difference between the levels of NS1 and NS2 mRNAs ($p>0.05$) at any time point. This has been clarified in page Results, page 7, line 176.

2. Functional assays to investigate the effect of selected NS2 interactions on virus infection/replication should be performed.

The whole manuscript has been modified and several new experiments, images and analyses has been added to clarify the importance of intact NS2 in infection by using selected NS2 associated proteins.

Our results indicate:

1) The NS2 splice donor mutation leads to significant increase in the amount euchromatin (H3K27ac) and in slight decrease in the amount of heterochromatin (H3K9me3) in comparison to wt or the NS2 splice acceptor mutant transfected cells. This indicates that the absence of functional NS2 leads to enhancement of cellular gene expression. These findings support the hypothesis that NS2 has a role in chromatin remodeling during infection (Figs 7 and S6).

2) NS2 performs functions that affect the quantity and distribution of proteins linked to DNA damage response. Notably, in contrast to MVM studies (Majumder et al. 2018) and our studies with the wt and NS2 splice acceptor s mutant showing close localization between replication centers and DDR proteins, H2AX and MDC1 were not associated with the viral replication centers in cells transfected with the NS2 splice donor mutant. Our findings suggest that NS2 is required for recruitment of H2AX and MDC1 to the viral replication region (Fig. 8).

3) Mutation of NS2 lead to changes in the establishment of replication centers. We showed that mutation in the splice donor site of the NS2 led to formation of multipole small viral replication center foci instead of the large seen in wild type virus-infected cells at late stage of infection (Fig 9).

All Figures and results of NS2 mutant data has been renewed, and a new chapter and figure of establishment of replication centers have been added We have also summarized the findings of the original study where the NS2 mutants were characterized and the effect of the mutations on viral infection was studied (Figures 7, 8, 9, S6 Results, pages 14-18, lines 360-469).

Majumder et al. 2018, https://pubmed.ncbi.nlm.nih.gov/30028293/

3. Fig. S1: The bright fluorescent spherical dots may be an artefact from NS2-EGFP overexpression as they cannot be found in the infected cells shown in Fig. S2.

The figure S1 shows the localization of the recombinant protein in NS2-EGFP transfected cells whereas Figure S3 (former Figure S2) represents nuclear distribution of NS1 in infected cells. The distribution of NS2-EGFP in Figure S2 is in line with the Figure IB showing non-infected NS2-EGFP transfected cells.

4. Line 234, (MKI67, BFDR <0.01). According to Table 1, BFDR is 0.04 (non-infected) and 0.02 (infected).

Expression BFDR <0.01 has been replaced by (MKI67; BFDR 0.02 in infected, and 0.04 in noninfected) (Results page 10, Line 252).

5. Line 375: "…findings demonstrate changes in the amount of heterochromatin in response to NS2 donor mutant transfection, suggesting that NS2 has a role in chromatin remodeling." This is not supported by the data. There is a significant difference between wt and non-infected cells but not between wt and the mutants (Fig. 6B).

We prepared new samples and analyzed further the virus induced changes in chromatin by analyzing euchromatin (H3K27ac) and heterochromatin (H3K9me3) in cells transfected with wt and NS2 mutant viruses. Our results demonstrated that the NS2 splice donor mutation leads to significant increase in the amount euchromatin (H3K27ac) and slight decrease of heterochromatin (H3K9me3) in comparison to wt or the NS2 splice acceptor mutant transfected cells. This supports the role NS2 in chromatin remodeling during infection.

The Figure and results have been renewed (Figures 7 and S6, results pages 14-15, lines 360-398).

Major Issues, Reviewer #3:

We are thankful to the Reviewer 3 for pointing out these important issues.

1. The relatively novel proximity-dependent biotinylation identification or BioID method is powerful. However, it requires the target protein (NS2 in this case) to accept and epitope tag and fusion with the promiscuous bacterial biotin ligase. The proximal targets identified were associated with a significantly modified, "mutant" form of NS2. What is evidence demonstrating that the modified NS2 protein remains functional and localizes in an identical manner to the unmodified protein? Similarly, although the N- and C-terminal fusions of EGFP to NS2 showed similar localizations after transfection, how does this compare to the distribution of the unmodified NS2 protein?

This is an important point. Appreciating the Reviewer's comment, we have added a new Figure 1C. This image shows that the HA- and BirA*-tagged NS2 construct (HA-BirA*-NS2, detected by anti-HA Ab) shows relatively similar homogenous nuclear localization as wt NS2 protein (detected by anti-NS2 peptide serum) in infected cells (see a new Figure S2). The NS2 is also sometimes localized into the nucleoli in the infected cells (Fig. 1D and S2) The text and figure legend has been modified accordingly (Fig 1C, Results, page 7, lines 191-199).

2. The final statement of the results, "These findings imply that NS2 might have a role in viral replication at cellular sites of DDR," implies that the DNA-damage repair proteins are associated with bona fide sites of cellular DNA damage. Since the spurious and widespread activation of DDR proteins may be a hallmark of many virus infections, this statement should be more carefully supported or appropriately modified.

Appreciating the Reviewer's concerns, we have removed this claim and instead added a modified statement at the end of the abstract. Please see subsection "our results provide insights into potential roles of NS2 in controlling chromatin remodeling and DNA damage response during viral replication. (Abstract, last sentence).

3. The statistical method used to evaluate difference among spots counted by the PLA assay may be inappropriate. Nonetheless, this is unlikely to affect the conclusion or significance. Specifically, Dunnett's method is an appropriate means of comparing the means of multiple groups against a single control. The comparison to a control group is appropriate. However, a more appropriate analysis for count data would be a Poisson regression or Dunnett's method applied to suitably transformed count values.

After a careful examination based on the reviewer's comment, we agree that Dunnett's test is not optimal for analyzing count data with low count values. To improve the analysis, we used Poisson regression to model the data. The Poisson regression showed that in all the other groups the count number was significantly higher than in the control group. Figures 5 and 6 as well as the description of the statistical analysis (Results page 13, line 343, Material and methods, page 29, line 760) have been modified accordingly.

Major Issues, Reviewer 4:

While the studies are meticulously performed and extensively controlled, several aspects of this study are puzzling. First is the surprisingly large number of apparent partners of such a small viral protein, only 165 amino acids in length. This may be because the fusion actually triples the molecular weight of NS2, and probably allows the biotinylation domain to interact promiscuously with many proteins in fairly loose proximity to any authentic, directly-interacting partner of NS2.

The BioID experiment were carefully controlled to remove the background from the unspecific binders. Controls included a total of 16 runs of four different GFP-BirA* constructs, including NLS-GFP, which should tag nuclear pore components encountered by most cargo. This allowed removal of nonspecific interactors after statistical filtering (using BFDR 0.05 as a threshold value). This methodology was explained in detail in Liu et al. 2018. Furthermore, the data was created by integrating control experiment data from CRAPome contaminant repository (Mellacheruvu et al 2013). In addition, BioID studies were done in Flp-In T-REx 293 cells to avoid artefactual interactions caused by overexpression of the NS2 (bait) protein. The large number of NS2 associated proteins could be explained by BioID detection of stable, transient and/or proximal interactions (Roux et al. 2012, 2013). These interactions can be either physical or functional. The reviewer´s comment about NS2 association with proteins proximal to NS2 interactor could be correct and therefore other biological methods to support interactions with representative proteins of BioID analysis were essential.

This has been clarified in the text (Materials and methods, page 24, lines 634-654).

Refs.
Liu et al. 2018, https://www.nature.com/articles/s41467-018-03523-2
Mellacheruvu et al. 2013, https://www.nature.com/articles/nmeth.2557
Roux et al. 2012, https://pubmed.ncbi.nlm.nih.gov/22412018/
Roux et al. 2013, https://pubmed.ncbi.nlm.nih.gov/24510646/

The proximity ligation technique also does not measure direct molecular interactions. Unfortunately, the authors do not pursue any more biochemical approaches, such as co-immunoprecipitation, to confirm direct, specific binding between NS2 and any of the identified proteins in the study.

PLA Proximity Ligation Assay was chosen to detect interactions with BioID identified proteins associated to NS2 in cells. Besides the quantitative analysis of associations (at distances < 40 nm) PLA demonstrates the intracellular localization of association sites even in single cell level. For example, PLA analyses demonstrated proximal localization of PLA signals and the nucleoli. In addition to PLA analyses of NS2-EGFP transfected cells, we have added a Figure 6 to confirm wt NS2 interactions with with BioID identified proteins in infected NLFK cells. The text has been modified accordingly (Results, page 12-14, lines 312-358).

Likewise, while the authors present an exhaustive discussion of how these possible interactions might be involved in CPV infection, the biological relevance of any of the identified associations is unclear. Indeed, it is puzzling why such an apparently interactive protein does not seem to play an essential role in CPV infection. For instance, the original paper describing CPV NS2 (ref 6), describes the elimination of the full-length protein by the introduction of in-frame termination codons in the NS2 ORF. This study found no significant effect of knocking out NS2 on the course of CPV infection of either cells in culture or dog tissues in vivo.

The original paper from Colin Parrish`s group (Wang et al 1998) showed that viral capsid protein (VP1/VP2), capsid and viral DNA production were all significantly decreased in cells transfected with NS2 splice donor mutant but not in in cells transfected with splice acceptor mutants. The production of capsid protein in the splice donor mutant transfection was less than 1/10 of the wt and other acceptor mutants examined. The splice donor mutant replicated very inefficiently and formed only very low levels of viral DNA and virus. Therefore, the in vivo infection of dogs was examined by inoculation only with one of the splice acceptor mutants not with the splice donor mutant. This shows that some key functions essential for viral replication are most likely removed from the NS2 splice donor mutant. We therefore believe that NS2 splice donor mutant is a very useful tool to support our studies of NS2 function.

Our most important findings from experiments performed with NS2 mutants support BioID results demonstrating that NS2 is associated with proteins linked to chromatin organization and DDR are following:

1) Intranuclear amount euchromatin is the altered in the absence of intact NS2

Our studies show that the NS2 splice donor mutation leads to significant increase in the amount euchromatin (H3K27ac) and slightly lower amount of heterochromatin (H3K9me3) in comparison to wt or the NS2 splice acceptor mutant transfected cells. This shows that the absence of functional NS2 leads to enhancement of cellular gene expression. This suggests that NS2 has a potential role in regulating host gene expression and chromatin remodeling during infection.

2) NS2 performs functions that affect the quantity and distribution of proteins linked to DNA damage response.

David Pintel´s studies with minute virus of mice demonstrated that DNA damage proteins localize to the periphery of MVM viral replication centers (Majumder et al 2018). Recruitment of H2AX and MDC1 to the replication region is required for the development of the replication centers. Notably, in contrast to MVM studies and our studies with wt and NS2 splice acceptor mutant showing close localization between replication centers and DDR proteins, H2AX and MDC1 were not associated with the viral replication centers in cells transfected with the NS2

splice donor mutant. Our findings suggest that NS2 is required for the normal progression of the DNA damage response in infection, and the mutation of NS2 splice donor site has most likely an effect on the recruitment of H2AX and MDC1 to the viral replication region.

3) Mutation of NS2 lead to changes in the establishment of replication centers

We showed that mutation in the splice donor site of the NS2 led to formation of multipole small viral replication center foci instead of the large centers seen in wild type virus-infected cells at late stage of infection.

The whole manuscript has been modified to clarify the impact of our main findings. All Figures and results of NS2 mutant data has been renewed, and a new chapter and figure of establishment of replication centers have been added (Figures 7, 8, 9, S6 Results, pages 14-18, lines 360-469).

Refs
Wang et al. 1998, https://pubmed.ncbi.nlm.nih.gov/9454701/

Part III – Minor Issues

Reviewer #1

Line 354: The magnitude of these differences are so marginal that it is not reasonable to make this conclusion without corroborating evidence. Also if the acceptor mutant does not make NS2 (and we need to know this), how can such an effect be attributable to NS2?

We analyzed further the virus induced changes in chromatin by using analyses of euchromatin, identified by H3K27ac, in cells transfected with wt and NS2 mutant viruses. Our results demonstrated that the NS2 splice donor mutation leads to significant increase in the amount euchromatin (H3K27ac) and slight decrease in the amount of heterochromatin (H3K9me3) in comparison to wt or the NS2 splice acceptor mutant transfected cells.

In the original paper from Colin Parrish`s group (Wang et al 1998) NS2 mutants were produced by introducing termination codons into the NS2 coding sequence of the infectious clone. In NS2 mutants, termination codons located to a site upstream of the common NS1/2 splice donor or downstream of the NS2-specific splice acceptor. Depending on the site of the termination codon most likely different sized NS2 proteins are produced. Although, characterization of mutants by immunoprecipitation analyses (antibodies against the NS2 C-terminus), showed that both mutants were unable to produce intact NS2 protein (Wang et al 1998), it is likely that the NS2 splice donor mutation leads to more drastically shorted protein than the splice acceptor. This is also supported by our experiments showing that the splice acceptor mutant is acting very similarly with the wt infectious clone (Figures 7, 8, 9, S6).

The Figure and results have been renewed (Figure 7 and S6, results pages 14-15, lines 360-398).

Refs.
Wang et al. 1998, Virology; https://pubmed.ncbi.nlm.nih.gov/9454701/

Line 363 and 365: “acceptor mutant” rather than “acceptor donor mutant”?
These mistakes have been corrected in results.

It is not always clear which experiments use NLFK cells and which use HeLa. Are there any concerns using HeLa cells to examine NS2/DDR protein connections?

All manuscript and figure legends were checked, and cell lines used were added if they were missing (results and all figure legends).

We are aware of potential heterogeneity and chromosomal instability of this HeLa cell line. However, Hela cells are one of the most popular cell lines in biomedical and cell biological research. It has also been widely used in recent high-quality DDR studies. Importantly, it is efficiently infected by the CPV and we have monitored the progression of infection in several of our previous studies. Moreover, many commercial antibodies essential for our immunolabeling studies recognize human proteins but fail to recognize feline proteins present in Norden feline kidney cells (NLFK).

Minor Issues, reviewer #2

1. Fig. 1C legend should be: …NS2 (green) and nucleolin (red).

Thank you, this has been corrected.

2. What cells were used for the BioID assay? Please indicate

BioID studies were done in the Flp-In T-REx 293 cells. This information has been added to the text and a figure legend (results, page 8, line 210, figure legend 3)

3. Line 318: In what sense did the intensities (spectral counts) correlate with the numbers of PLA foci? How was this determined. Please clarify.

We apologize for unsuitable diction since no actual correlation was measured. The average number of nuclear PLA foci of NS2 together with SMARCA5 and macro-H2A.1 was consistent with the high-confidence interactions that were observed by BioID. The expression correlation has been replaced.

4. Line 604: “…annotation chart of NS2 interactors (Table S1).” Should this be Table S2?
Yes, this has been corrected (materials and methods, page 25, line 654).

5. In the text and figures non-infected sometimes means NS2-EGFP transfected (e.g. Figs. 2, 3), sometimes it means non-transfected (e.g. Figs. 6, S4, S5, 7). This is confusing.

We apologize for the confusion. The whole manuscript and specifically figures and figure legends were checked and clarified

6. Fig. S2: Nuclear staining is missing.

The nuclear stain has been added to the Figure S2.

7. Fig. 5A: the representative image of the macro H2A.1 staining has roughly 70 foci, not 135+/-12 as indicated in the results (line 314).

The representative images of stained cells show single confocal optical sections of cells whereas number of PLA signal foci per nucleus was analyzed from 3D reconstruction of confocal images. This has been clarified in the text and figure legend (Results page 13, line 340; figure legend 5).

Minor Issues, reviewer #3:

What exactly does the phrase "emergence and distribution of DNA damage effector proteins" mean? Perhaps a more direct statement of the concept could be used in the Abstract instead of this somewhat obscure description. The phrase in the abstract, "studies with NS2 mutants indicated that NS2 performs functions that affect the quantity and distribution of DNA damage effector proteins" is also vague and conveys little information. Rather than this vague description, perhaps specific and succinct details could be provided.

We agree that the expression and the sentence were somewhat obscure. Appreciating the Reviewer's suggestion, we have modified the expression and sentence (Abstract, lines 44-45).

Another example of unnecessarily vague writing is in the Author Summary. The statement"serves a previously undefined important function in essential nuclear processes that occur during viral infection" does not convey meaningful specific information. Rather than simply state these vague assertions, tell the reader what they are.

We agree. The modified sentence is "Our studies indicate that NS2 serves a previously undefined important function in chromatin modification and DNA damage responses (Author summary, page 3, line 63-65).

The network graph presented in Fig 2A is nearly indecipherable and of marginal utility. It seems a textual presentation with this information (of less complexity than Table S1) could be more valuable.

This image shows the schematic representation of 48 NS2 associated proteins identified using BioID. Nodes represent unique proteins identified and categorized as chromatin modification DDR proteins according to the function based on Gene Ontology (GO) annotation analyses of biological processes, Entrez Gene, UniProt and primary literature. In addition, the image shows which associations are found in the absence and presence of infection. This presentation form or its modifications are generally used in presenting BioID data. We agree that it is a challenge to create a clear presentation of this data. However, we feel that this presentation together with tables (1,2,3) and the textual presentation focusing on NS2 associated key factors in chromatin remodeling and DDR are needed to support our findings.

Line 175: The studies in MVM (reference 70) are cited as being able to "account for the relatively small amount of nuclear NS2 detected in CPV infection." If this is referring to a CPV infection, more information on the "small amount of nuclear NS2" is required to justify this statement.

Thank you for pointing this out. This statement was referring to a MVM infection not CPV. This has been corrected (results, page 7, line 181).

Line 187: What is the basis for stating that "many" DDR factors reside in nucleoli? Is it more than the nucleoplasm proper? Qualification of this statement and a reference is needed.

We agree that this statement was somewhat unfounded. Appreciating the Reviewer's suggestion, we have modified this expression and added the references (results, page 8, lines 202-203).

Results of the associations measured by "BioID" are introduce at line 192 with no discussion on the method, its strength and its limitation. It would be appropriate to include some preamble on this uncommon method such as was done for the PLA method on at lines 304-308.

Introduction to BioID has been added to results (results, page 8, lines 206-209).

I am curious as to why the strongest interaction detected was with Ki-67? Is there any evidence pointing to the mitotic role of Ki-67 versus the role it plays in interphase cells?

In addition to the role of Ki-67 as an organizer of heterochromatin in the interphase cells (Verheijen et al. 1989, Sobecki et al. 2016), it has been shown that Ki-67 is involved in the spatial organization and motility of mitotic chromosomes (Cuylen et al. 2016). Ki-67 acts also as a promoter of chromosome clustering before the nuclear envelope reformation at late stages of mitosis (Cuylen-Haering et al. 2020).

In general, parvoviral NS2- Ki-67 interaction is an important point and it will be exciting to pursue this topic in the future.

Refs.
Sobecki et al. 2016 eLife; https://elifesciences.org/articles/13722
Verheijen et al. 1989 J Cell Sci; https://pubmed.ncbi.nlm.nih.gov/2689459/
Cuylen et al. Nature 2016; https://pubmed.ncbi.nlm.nih.gov/27362226/
Cuylen-Haering et al. Nature 2020, https://pubmed.ncbi.nlm.nih.gov/32879492/

Although informative, the text describing the identification of various DDR factors associated with NS2 (lines 258 - 289) seems to be more of a discussion of the DNA-damage response than a statement of the results. Nonetheless, a simple recitation of associated factors would be of little value. On balance, this is appropriate but could possibly benefit from some editing to reduce the amount of text.

We agree that the chapter "NS2 is associated with proteins linked to chromatin organization" should have been presented in a more compact form. The whole chapter has been modified, and the amount of text significantly reduced (results, pages 9 and 10, lines 238-267)

Were any other factors influencing the choice of SMARCA5, macro-H2A.1 and SSRP1 as targets for the PLA other than availability of antibodies. If so, it may be helpful to provide the rationale.

These NS2 interacting proteins represented chromatin modeling (SMARCA5) and DDR (macro-H2A.1, SSRP1) functions (see Fig. 4). Moreover, their association with NS2 was detected both in noninfected and infected cells. This has been clarified in the text (results, page 12, lines 314).

The statement at line 323, "It is possible that NS2 association with SSRP1 is transient as it is detected clearly by BioID whereas hardly by PLA," is likely but it may help the reader to justify this with a reference supporting the notion that transient interactions are better detected by BioID.

BioID allows to probe stable, transient and proximal interactions of the protein of interest (Roux et al 2012, 2013). In BioID provides a recording of all interactions (biotinylation of proximal proteins) occurring during the 20 hours incubation period (after tetracycline induced expression of HA-BirA*-NS2 and biotin addition). In comparison, PLA shows interactions of the prevailing situation at the time of the cell fixation. This was clarified and references were added in the text (results, page 8, line 208; Materials and methods page 24, line 639).

Refs.
Roux et al. 2012, https://pubmed.ncbi.nlm.nih.gov/22412018/
Roux et al. 2013, https://pubmed.ncbi.nlm.nih.gov/24510646/

Why is the NS2 acceptor mutant virtually wild-type? Are alternative splice acceptors being used? If the use of the NS2 splice acceptor mutant as wild-type is standard practice, it would be helpful to cite a reference for this. Otherwise, an explanation of why this "mutant" is not mutant is appropriate.

NS2 mutants were produced by introducing termination codons into an infectious clone (Wang et al. 1998). Characterization of mutants by immunoprecipitation analyses (antibodies against the NS2 C-terminus), showed that both mutants were unable to produce intact NS2 protein (Wang et al. 1998). However, the length of NS2 produced by mutants depend on the location of the termination codon. The donor mutant produces NS2 which is drastically shortened. Transfection with this mutant leads to significantly decreased production of viral capsid proteins, capsids, and viral DNA in comparison to wt infectious clone. In contrast, the NS2 splice acceptor mutant produces longer NS2 which allows approximately equal production of viral capsid proteins, capsids, and viral DNA to those produced wt infectious clone. We did not use alternative splice acceptors mutants in this paper. We would also like clarify that NS2 splice acceptor mutant was not used as a wild type (wt) construct instead we used the infectious clone (Parrish 1991).

We have modified the text to clarify characteristics of NS2 mutants (results, page 14, lines 360-376.

Refs.
Wang et al. 1998, Virology; https://pubmed.ncbi.nlm.nih.gov/9454701/
Parrish 1991, Virology; https://pubmed.ncbi.nlm.nih.gov/1647068/

The measurement of "correlations" of various proteins localized in Fig. 7 are discussed at line 404 without any definition of what is meant by "correlation." This should be added.

We have replaced the correlation analysis with the more suitable fluorescent intensity line profile analyses. The whole chapter (page 15-16, lines 400-469), figure 8 and figure legend 8 have been modified.

Required corrections indicated by line numbers

90: space needed in "thenuclear"
273: "chaperon" is misspelled
343: space needed in "thesplice"

These changes have been made.

347: the phrase "in the splice donor mutant virus transfected cells" requires hyphens to connect adjectival nouns.

We used expression “in the cells transfected with the splice donor mutant virus” (results, pages 14-16).

353: "donor mutant was transfected; in contrast it was lower compare" requires editing (a comma, for one thing)

This sentence was removed when new data was added.

417: Rephrase "This could propose that…"

This was replaced by “This suggests that”.

Reviewer #4:

(No Response)
