# Supplementary material for: Parvovirus nonstructural protein 2 interacts with chromatin-regulating cellular proteins
Source: PLoS Pathog. 2022 Apr 8;18(4):e1010353. doi: 10.1371/journal.ppat.1010353 (PMC9020740; doi:10.1371/journal.ppat.1010353)
Supplement: Attachment — Submitted filename: Revision letter .pdf [file ppat.1010353.s012.pdf]

PLOS Pathogens
Editors

Karl Münger
Kasturi Haldar
Michael Malim

March 5th, 2022

Dear Editors,

Please find enclosed the revised manuscript "*Parvovirus nonstructural protein 2 interacts with chromatin-regulating cellular proteins*" by Salla Mattola et al.

We have now addressed all the points raised by the reviewer 1 and 4 as outlined in our response letter. Based on their suggestions, appropriate changes have been introduced to the manuscript. We trust we have been able to address their concerns and hence to improve our work.

We hope our manuscript is now suitable for publication in PLOS Pathogens.

Yours sincerely,

Colin Parrish, Maija Vihinen-Ranta et al.

Department of Biological and Environmental Science
University of Jyväskylä, Finland
Phone: +358 400 248 118
Email: maija.vihinen-ranta@jyu.fi

Response to comments of reviewer #1 and 4

Reviewer #1

CPV NS2 is an enigma. In this revised manuscript the authors convincingly demonstrate that NS2 interacts with a number of proteins involved in chromatin remodeling and the cellular DNA damage response. This is valuable information but essentially descriptive. The authors analyze mutants of NS2 to make a functional connection; however, questions remain concerning the use of the NS2 mutants chosen.

To determine the biological relevance of the interactions, and the functional significance of NS2 in this regard, the authors rely on analysis of transfected CPV plasmid clones that bear mutations of NS2 which have been previously characterized (Wang, 1998). In those published studies, mutations affecting the R2 splice donor or acceptor, as well as termination mutations (one, at nt 2033, not far downstream of the R2 splice acceptor), were analyzed. None of these generated NS2 molecules recognizable by antibodies directed to the NS2-specific C-terminal exon; however, all showed wild-type phenotypes, except for the splice donor mutant, which was substantially defective. The same splice donor and acceptor mutants were used in this study. As evidence of a specific NS2 connection the authors show that the splice donor mutant displayed differences in the assays examined, while the splice acceptor mutant, also devoid of the NS2-specific exon, did not. It is important to note that the N-terminal exon of NS2 is shared with NS1. How to square those results with the results presented here?

In their rebuttal to previous critique of this point the authors suggest that: "some key functions essential for viral replication are most likely removed from the NS2 splice donor mutant", and "the NS2 splice donor mutation likely leads to drastically shorter protein than the splice acceptor." Little is known about what products are actually generated by the splice donor or splice acceptor mutant, and cryptic splicing was detected for both at levels not reported (Wang, 1998). The previously identified termination mutant, however, would be predicted to generate an NS2 molecule truncated merely 10 amino acids following splicing into the NS2-specific C-terminal exon.

There seem to be a limited number of possibilities that would support the authors arguments. One is that the splice donor mutation leads to accumulation of very little R2 at all, and that, although unlikely, a certain amount of the independent NS1/2 shared exon is required for wild-type "NS2" activity. Alternatively, since the 2033 termination mutant was previously shown to be wild-type, perhaps the 10 amino acids left in the NS2 unique exon of that mutant affords its function and is the bit the authors are referring to. (Cryptic splicing in the splice acceptor mutant joins significantly downstream at nt 2099 and has a larger NS2-spcific exon piece.) Finally, it also remains possible that aberrant splicing of the donor mutation (which uses a cryptic donor 4 nts downstream of the wildtype site) results in a dominant negative NS2 molecule responsible for the effects seen.

I suppose the authors' rationale for using these mutants can be accepted – even though the precise nature of their NS2 defects are not clear. But the possibilities mentioned above pose a nagging, obvious, open question coloring the manuscript which need to be more

fully discussed somewhere in the manuscript, and hopefully addressed experimentally at a later date.

Reviewer #4

This is an extensive, sophisticated and meticulously performed study of interactions between host cell proteins and what the authors represent as the NS2 gene product of Canine Parvovirus (CPV). The major concern that remains for this reviewer is whether these interactions are truly NS2 specific. The reason for this concern comes from a consideration of a previously published analysis of CPV NS2 mutants.

The NS2 gene of CPV encodes a 165 amino acid polypeptide, of which the N-terminal 87 amino acids are contributed by genomic sequence common to NS2 and the N-terminus of NS1, the major replicative non-structural protein of the virus. In NS2, this common N-terminal sequence is linked by mRNA splicing to a further 78 amino acids encoded in an alternative reading frame colinear with downstream sequences of the NS1 gene. The original paper characterizing CPV NS2 (Wang et al., 1998 – Ref 6), describes the analysis of five mutants designed to disrupt the expression of NS2 without affecting the amino acid sequence of NS1. Of these, three translation termination mutants and a splice acceptor mutant showed no discernable phenotype with respect to viral DNA replication, structural protein synthesis, capsid assembly and production of infectious virus, and where tested, infection of dogs, leading to the conclusion that most, if not all, of the C-terminal NS2-specific sequence is not required for any currently measurable facet of the viral infectious cycle.

This is in marked contrast to the results of similar experiments with NS2 mutations in the genomes of rodent parvoviruses, predominantly Minute Virus of Mice, where abrogation of the second exon of NS2 leads to abortive infection in normal cells of the host species and abolishes pathogenesis in infected host animals. One of the five CPV mutations, that affecting the donor splice site, does exhibit a phenotype of impaired viral DNA replication and capsid protein synthesis. This puzzled Wang et al., who stated that "Analysis of the effects of altering the splice donor or acceptor sequences was complicated by the fact that in each case an alternative sequence used to splice the message was detected by RT-PCR." Despite this warning, and the similar concerns of reviewer 1, the authors continue to emphasize that they consider the splice donor mutant to be a simple NS2 null mutant, with an interesting phenotype representing solely the absence of NS2. It seems extremely unwise to ignore the strong indications from the other mutants studied in the Wang et al., paper that the C-terminal 78 amino acids of NS2 are dispensable, and thus are extremely unlikely to harbor interaction domains of any relevance to the viral life cycle.

This consideration suggests the distinct possibility that the biologically significant interactions revealed in the current study are properties solely of the 87 N-terminal amino acids of NS2 – and therefore also of NS1. Thus, it seems quite possible that the interactions revealed in this manuscript are properties of NS1, or that NS2 acts as an N-terminal domain of NS1 liberated from its nickase, helicase and transcriptional activator functions by mRNA splicing.

Our response to both comments (which address similar issues):

We thank Referees 1 and 4 for their knowledgeable comments and help in improving this manuscript. We have read the additional comments carefully and have tried to interpret those relative to any additional changes in the manuscript presented.

General response: Our understanding of these review comments is that the issues raised would be more applicable to future studies where the more subtle effects could be explored – since those would require production and analysis of additional NS2 mutants, and the analysis of the smaller mRNA and identification and analysis of possible smaller protein forms. As the NS2 protein itself has a relatively short half-life, we assume that any artificially truncated versions would be even more transient in form, and therefore hard to study – and therefore are likely outside the scope of this study.

Specific responses: We appreciate that the role(s) of NS2 in the parvoviruses are complex and are still not well understood, and their expression and sequences also clearly vary in different viruses, in the few cases where they have been examined. Most of the previous detailed work on NS2 in parvoviruses was conducted with MVM, which does have a distinct expression pattern with two donors and two acceptors, which allowed for more spliced isoforms and additional strategies for modifying expression. In the MVM studies the presence of the C-terminal domain was assumed to be a requirement of the full NS2 function, and no role of a possible truncated (i.e. mainly NS1-derived) form is known. In MVM the NS2 mutant viruses showed clear but subtle effects, and some of the phenotypes were only revealed in specific host cell contexts. The previous data on the CPV NS2 was intended to compare the effects on that particular virus, and showed that despite the retention of the NS2 ORF and of the splice machinery, the C-terminal domain could be largely deleted with modest effects on the viral replication in tissue culture cells and animals.

We therefore agree that the reviewers that splice donor mutant may not be completely NS2 null mutant, and we have added the sentences "However, the interpretation of mutant-induced effect on viral life cycle was complicated by RT-PCR characterization which revealed that alternative sequences used to splice the message RNA were present in both mutants [Wang et al. 1998]. It should be also noted that, that the N-terminal end of NS1, which share a common N-terminal domain with NS2, might be produced in some circumstances, and might play a role in virus-induced chromatin remodelling, although that remains to be defined. (Results page 14-15, lines 348-360).

Studying any role of CPV NS1 N-terminal domain in chromatin remodelling is challenging. Our studies using whole NS1 containing constructs for proximity-dependent biotin identification (BioID) have so far been hampered by cytotoxicity of the NS1. The future experiment suggested by reviewer #4, the BioID assay using N-terminal fusion of the BirA* tag with NS1/2 with and without the splice donor sites could be potentially informative in this context.

Ref. Wang et al. 1998, https://pubmed.ncbi.nlm.nih.gov/9454701/

Reviewer #4

The probability that the splice donor and acceptor mutants activate cryptic acceptor and donor splice sites, respectively, should be examined by sequencing of RT-PCR products of appropriately spaced primers. This would reveal whether either of these mutations result in the expression of aberrant forms of NS1.

This is an excellent idea - these NS2 mutants or other additional mutants may be usable for future studies but would require a significant investment of time and resources, and as seen in the other studies of NS in CPV, MVM, and other parvoviruses, are likely to need significant additional characterization.
